# Leading by learning: A lifelong commitment to leadership programme

**DOI:** 10.4103/0971-3026.44521

**Published:** 2009-02

**Authors:** Rajesh Gothi

**Affiliations:** Diwan Chand Aggarwal Imaging Research Centre, New Delhi-110 001, India

In the first place, we should be thankful for being a part of this stream of medicine. Radiology is a perfect blend of medicine, surgery, and their related specialties, a feast for academicians and technocrats, a photographer's delight, a challenge for the administrator, a friend of clinical colleagues, and an advisor to the patient. Need we ask for more!

The answer is ‘yes.’ We must ask for a burning desire, a passion to know more and to keep up with the ever-evolving knowledge emerging from fresh experience, new insights, and the advancements in technology.

We embark on our professional journey with the basic information acquired during our residency program.[1] This ‘hard core’ of medical knowledge, learnt at the undergraduate and residency level, is forever shifting and changing.[2]

The end of residency marks the beginning of real life. The responsibilities that come with it take up a good portion of our time, both in our office practice as well as in our work in hospitals. The demands made on us may not necessarily coincide with our learning needs.[2]

In this talk, I will deal with the post-residency period of life—the period when we step into the outside world and accept the many responsibilities coming our way, all the while trying to keep abreast of the latest developments, without forgetting the previously acquired knowledge.

To begin with, we have to admit and recognize that medicine is too vast a subject for anyone to master in one lifetime. Countless men and women before us have spent their lives unraveling the mysteries of the human body and healing people, they have enriched us with their experience and led us to where we are. Yet, there is an unfathomable depth that needs to be plumbed, distant horizons that may for ever remain beyond human reach. New frontiers will be created by man's unending quest for physical upliftment. In this scenario, we must not forget that no matter how good we are, no matter how dedicated and brilliant we are, our contribution to the profession will remain miniscule at best.

We must remember that medical treatment, by itself, contributes to the health of the nation in important but, nevertheless, small way. The major contributors to good health and overall well-being are immunization, nutrition, safe drinking water, hygiene, and emotional health.[3] Once this is understood, then we will be able to clearly see our standing in the scheme of things and be able to lower our egos to sensible levels. But, having said that, I should also remind you that it is the small blocks that make up the building. In this context, I will recall the teaching of the Bhagwad Gita: ‘do your duty and leave the rest in His hands.’ Add to this the new-age mantra given by Nike: ‘Just do it,’ and we are ready for the process of lifelong learning!

We must learn constantly, for there is nothing which gives a greater high than the satisfaction of knowing more and more. Indeed, knowledge is life. To quote James Byrant Conant, ‘Each walk of life, each honest calling, has its own aristocracy based on excellence of performance.’ We must all strive as hard as possible to excel in our chosen field.

Something more about learning: learning is actually a form of meditation—it brings mental peace, improves well-being, and keeps one away from ill health! To quote Stephen Leib[4]:‘It does many things, like building relationships, fulfilling social obligations, personal advancement including staying ahead of competitors, outsmarting the peers, escaping from monotony and boredom, and keeping mentally fit.’[4]

Therefore, there is no doubt that our learning must go on as long as possible.

We come now to the question of how to go about it. Most of us, busy with our jobs and practice, are hard pressed for time, having to juggle numerous responsibilities during our working hours. Our learning also tends to become need based. Not all advancements are of use in our daily practice. Information that cannot be used does not hold our interest for long and is soon forgotten. We often do what is called ‘just-in-time learning,’ meaning accessing relevant information only as and when needed[1] rather than stacking every bit and piece of information into our memory bank. Added to this, are our own human limitations. We multitask, but forget that our vision and hearing are no longer what it once was. Many of us, including myself, suffer from attention deficit also, I often wonder how everyone else can concentrate on a lecture but I cannot!

We must device ways and means, somehow extricate ourselves from our other responsibilities, and find time to obtain information useful to us[4] and lock it away in our memory bank.[5]

With time, our learning becomes predominantly self-directed,[6] with a greater need to know why we should learn something.[7] We must remain focused on learning and remember the negative effects of not knowing.[5] Since the process of learning and getting trained is likely to be self-driven, the professional association we belong to should take enabling steps by providing us with learning modules and assessing the outcome of our efforts.

How else do we go about learning? We must search for our shortcomings and the challenges we face in our daily work, at the end of the day we must look back at how we have performed. From this introspection I, for one, learnt a new mantra: ‘caution, hesitation, and a small amount of self-doubt are actually good.’ It is better to be cautious than careless. So whenever there is even an iota of doubt, it helps to get back to the books, to surf the net, or to ring up a colleague.

Next, we must make it a habit to read for at least a short while every day. The reading can be from the Net or journals. Subscription to journals must be a part of our work expense. The journals we read should include at least one non-radiology journal. We should know what is happening outside our field. We must also read the medical news available on the internet or in reputed newspapers and magazines. It will give us a broader outlook and keep us in touch with the latest medical news.

We should consider teaching formally as well as informally. This will enable us to recall what we have learnt and also clarify our concepts. It would be best if we could find attachment with teaching institutions. If not, then we can participate in local meetings or form small study groups of like-minded persons, where we can blend education with socializing. It would be great if teaching institutions in this country can open up, ideally, they should make use of experts working outside the teaching hospitals, inviting them as guest lecturers. The type of diseases seen in community practice and the experience in office setups is different from that seen in teaching hospitals. Teaching institutes can also advertise their teaching programs on the net so that interested persons can attend their programs. If their teaching programs are good, their reputation will also grow. The aim should be to achieve an all-round improvement in the standard of education and not just to establish a few islands of knowledge in a sea of ignorance. Institutions, especially private ones, must make it a point to see that their consultants are actively involved in the two-way process of education—that is, teaching and learning. They should contribute in whatever way possible to build up excellence in their staff. Only then can institutions attain greatness. Having the latest machines or a five-star-hotel-like ambience is not enough!

The internet has been a great boon for those seeking to educate themselves at their own pace, balancing the responsibilities and the good things of life with education. There are several sites offering quizzes and reviews and dealing with the latest topics. These sites are easily accessible and one must make full use of them.

There are a few things that are not taught during residency programs or at the undergraduate level but are, nevertheless, very important. These are the soft skills. These skills will help to be a good teacher, to understand human behavior, be a good administrator, and to handle money matters. A good teacher learns first and then presents the material learnt in such a way that that the audience goes back fulfilled. This process of teaching comes with practice, dedication, and untiring effort. We should aim to become good communicators.[8]

In order to be successful in life a high emotional quotient is very important. Our attitude towards patients, their relatives, our colleagues, and the other staff is very important. Politeness, courtesy, eagerness to help, and the ability to listen patiently are not just empty words, success, respect, and job satisfaction come to those who make the effort to practice these niceties in their workplace. I have often noticed that many doctors in this country harbor a feudalistic attitude. They do not treat their junior colleagues and staff with courtesy. I remember one occasion when, during a meeting, an x-ray technician pleaded for nothing more than to be treated with respect. We may be under the impression that our behavior is impeccable but our faces and actions often betray our true feelings. We have to learn the art of controlling our emotions and creating a pleasant workplace. This aspect of education is very important, it is not taught in schools and colleges but it must be learnt at all cost.

As we go up in the hierarchy, we may also have to accept administrative responsibilities. The art of being firm yet kind and of being successful without being unethical comes with proper upbringing and with the exercise of self-restraint. Our endeavor must be to develop an efficient and disciplined yet cordial environment at our workplaces. A bit of spiritualism comes in handy in difficult times!

Our growth will bring responsibilities: we will be called upon to plan our departments, purchase equipments, negotiate with vendors, and deal with financial matters. A short course in administration and finance or an apprenticeship under an able administrator may be worthwhile.

Finally, always remember that there is life outside the workplace—we have not come into this world only to practice radiology! The satisfaction that comes with having lived life to the full should not be sacrificed at the altar of professional education. A proper balance between work and play is essential for a happy mind and a healthy body.

To conclude, education is for life.[9] Life-long learning will contribute to our happiness and good health. Our learning, and recall of things learnt, is likely to improve if we adopt problem-based learning.[10] We must take advantage of all available resources for learning and devise our own learning strategies, such as forming Web groups,[11] and take positive steps to attain excellence in our chosen field. Keeping ourselves up-to-date should help us improve our mental faculties, to ward off illnesses, and make us better professionals as well as leaders in our chosen field.
